# Physiological Properties of Three Pelagic Fungi Isolated from the Atlantic Ocean

**DOI:** 10.3390/jof9040439

**Published:** 2023-04-04

**Authors:** Eva Breyer, Salvador Espada-Hinojosa, Magdalena Reitbauer, Samantha C. Karunarathna, Federico Baltar

**Affiliations:** 1 Department of Functional and Evolutionary Ecology, University of Vienna, 1030 Vienna, Austria; 2 Center for Yunnan Plateau Biological Resources Protection and Utilization, Yunnan Engineering Research Center of Fruit Wine, College of Biological Resource and Food Engineering, Qujing Normal University, Qujing 655011, China

**Keywords:** ecological role, fungal isolates, marine fungi, metabolism, morphology, FF MicroPlate

## Abstract

Oceanic fungi are widely understudied compared to their terrestrial counterparts. However, they have been shown to be important degraders of organic matter in the global pelagic oceans. By examining the physiological characteristics of fungi isolated from the pelagic waters of the ocean it is possible to infer specific functions of each species in the biogeochemical processes that occur in the marine ecosystem. In this study, we isolated three pelagic fungi from different stations and depths across a transect in the Atlantic Ocean. We identified two yeasts [(*Scheffersomyces spartinae* (Debaryomycetaceae, Saccharomycetes, Ascomycota) and *Rhodotorula sphaerocarpa* (Sporidiobolaceae, Microbotryomycetes, Basidiomycota)], and the hyphae-morphotype fungus *Sarocladium kiliense* (Hypocreales, Sordariomycetes, Ascomycota), and conducted physiological experiments to investigate their preferred carbon uptake as well as their growth patterns under different environmental conditions. Despite their taxonomic and morphological differences, all species exhibited a high tolerance towards a wide range of salinities (0–40 g/L) and temperatures (5–35 °C). Furthermore, a shared metabolic preference for oxidizing amino acids was found among all fungal isolates. Collectively, this study provides relevant information on the physiological properties of oceanic pelagic fungi, revealing a high tolerance towards salinity and temperature changes, ultimately contributing to understanding their ecology and distribution in the oceanic water column.

## 1. Introduction

Fungi are ubiquitous in terrestrial, freshwater and marine habitats [1]. Despite their global importance, most previous mycological research focused on either terrestrial [2,3], freshwater [4,5], or sediment-derived fungi [6,7] outpointing the high undiscovered potential of oceanic pelagic fungi in terms of diversity and secondary metabolite production [8,9]. Additionally, many marine-derived fungi also occur in terrestrial and freshwater ecosystems. This emphasizes the remarkable adaptive capabilities of fungi but also generates the question of how many already described terrestrial species inhabit marine environments [8].

With these exceptional adaptive abilities, it is not surprising that, according to metagenomic analyses, fungi are present in all oceans, dominated by Dikarya [10]. Globally, marine fungi have also been isolated from different substrates and regions, including extreme habitats such as the deep sea [6], hypersaline anoxic basins [7], hydrothermal vents [11] and arctic sea ice and water [12].

Marine fungi exhibit a range of life strategies, which is attributed to their extensive presence in diverse environments. These strategies may involve distinct approaches for obtaining nutrients and energy, coping with environmental pressures, or adapting to different ecological niches. As saprophytes (e.g., *Malassezia* and *Cladosporium*), they are decomposing recalcitrant substrates, ultimately re-integrating them back into the food chain and hence making them accessible to other organisms [13,14]. Certain parasitic fungi from the Chytridiomycota group, such as *Rhizophydium* and *Chytridium* [15,16,17], are common parasites on marine diatoms. These fungi produce zoospores that are readily available to zooplankton, a process known as the “mycoloop” [4]. Recent research suggests that marine fungi dominate microbial biomass on bathypelagic marine snow [18]. In addition, pelagic fungi, particularly in the classes of Dothideomyctes, Eurotiomyctes, and Leotiomycetes, express a diverse range of CAZymes [19] and proteases [20] that degrade organic matter in the global oceans, indicating their active contribution to the cycling of major elements that make up marine biomass, both at global [19,20] and local scales [21].

Given their global distribution [10], active participation in the marine C-cycle [19] and N-cycle [20] and important role in marine food web dynamics [4], oceanic fungi are considered as being crucial participants in global marine biogeochemical processes [8].

According to Jones, et al. [22], due to next generation sequencing (NGS), lots of sequences have been detected, arising from fungal species that have never been observed in cultures. This leads to a gap of knowledge between the uncultured “dark fungi” and well-documented species descriptions [1,23,24]. Taking into account their important role as secondary metabolite producer and their relevant contribution to the remineralization of organic matter in the oceans, there is an urgent need for identifying and physiologically characterizing marine fungal isolates.

To deal with the aforementioned issues, we took the following steps: isolating three fungi from the marine pelagic environment, identifying them, and examining their morphology, growth physiology, and carbon substrate uptake by using FF MicroPlates^TM^. With this information, we aimed to expand our understanding of the distribution, the physiology and the ecological properties of pelagic fungi in the ocean.

## 2. Materials and Methods

### 2.1. Sample Collection and Isolation of Marine Fungi

**Figure 1:**
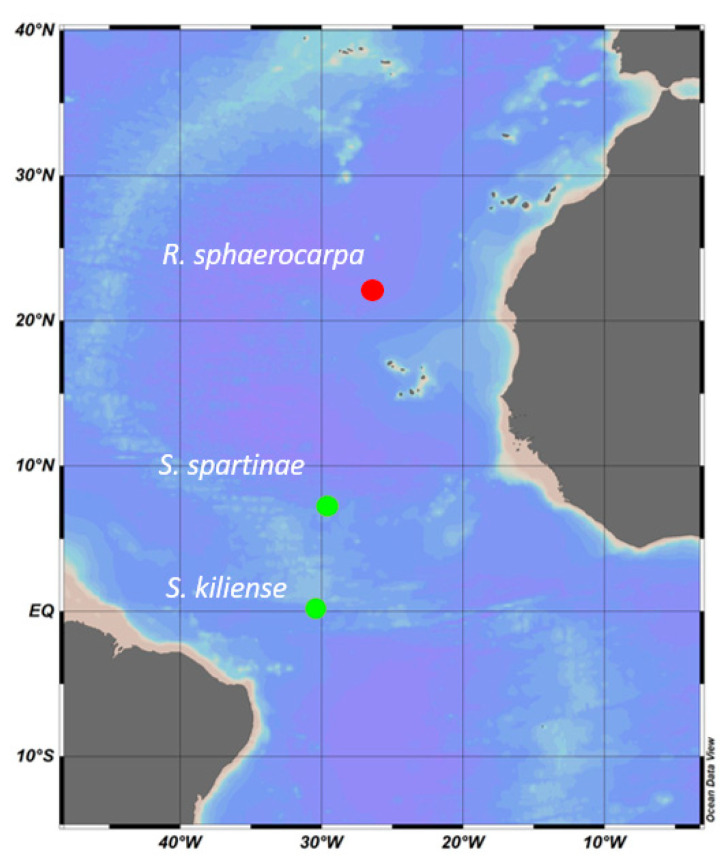
**Figure 1**. The three sampling stations where the fungi were isolated during the ANTOM-1 Cruise in 2020/21 (green dots) and the Poseidon Cruise in 2019 (red dot).

Pelagic fungi were collected during the ANTOM-1 Cruise in 2020/21 and during the Poseidon Cruise (2019) on board the RV Sarmiento de Gamboa (Figure 1, Table 1). 

Seawater samples were collected with a CTD rosette equipped with 12-L Niskin bottles. The sampling depth ranged from 5 to 2000 m during the ANTOM-1 Cruise and from 5 to 6197 m during the Poseidon Cruise. When collecting the fungal isolate during the Poseidon Cruise, unfiltered seawater from the respective sampling depth was pipetted directly onto the solid agar media plate (media concentration see below). To collect fungal isolates during the ANTOM-1 Cruise, around 500 mL of seawater per sampling depth were filtered onto a combusted GF/F filter. Afterwards, the filter was inoculated on a solid agar media plate containing (g/L): 1 g glucose, 1 g peptone, 1 g yeast extract, 1 g starch, 20 g artificial sea salts, 15 g agar and 0.5 g chloramphenicol. The agar plates were incubated at environmental temperatures and checked for growth on a daily basis. Once individual colonies started to emerge, they were separated onto distinct plates, and subsequently re-plated until a single species was completely isolated. 

### 2.2. Sequencing of the Fungal Isolates

To identify the species, fresh agar plates were prepared from all isolates and then allowed to grow at room temperature. The solid agar media contained (g/L): 20 g agar, 10 g glucose, 5 g peptone, 3 g yeast extract, 3 g malt extract, 35 g artificial sea salts and 0.5 g chloramphenicol. Afterwards, the cultures were sent to the Westerdijk Fungal Biodiversity Institute Identification Service (Utrecht, Netherlands, https://wi.knaw.nl, accessed on: 15 March 2023) for species identification. There, after arrival, the strains were cultivated on malt extract agar (MEA) and dichloran 18% glycerol agar (DG18). The DNA was extracted from MEA after an incubation period of 3 days in the dark at 25 ˚C using the Qiagen DNeasy Ultraclean™ Microbial DNA Isolation Kit. For all strains, fragments containing the Internal Transcribed Spacer 1 and 2 regions including the 5.8S rDNA (ITS) and the large subunit region D1 and D2 (LSU) were amplified and sequenced. The primers used for the ITS region were: LS266 (GCATTCCCAAACAACTCGACTC) and V9G (TTACGTCCCTGCCCTTTGTA) and for the LSU region LR0R (ACCCGCTGAACTTAAGC) and LR5 (TCCTGAGGGAAACTTCG). Furthermore, in the case of *Sarocladium kiliense*, a partial fragment of the actin gene (Act) was amplified and sequenced. The primers used were Act512F (ATGTGCAAGGCCGGTTTCGC) and Act-783R (TACGAGTCCTTCTGGCCCAT). The PCR fragments were sequenced in both directions with the primers used for PCR amplification using the ABI Prism^®^ Big DyeTM Terminator v. 3.0 Ready Reaction Cycle sequencing Kit. Samples were analyzed on an ABI PRISM 3700 Genetic Analyzer and contigs were assembled using the forward and reverse sequences with the program SeqMan from the LaserGene package. The sequences were compared on GenBank using BLAST and in the in-house sequence database of Westerdijk Fungal Biodiversity Institute (Table 1).

### 2.3. Morphological Observations with Epifluorescence Microscopy

For morphological observations, the fungal cultures were grown in liquid media containing (g/L): 2 g glucose, 2 g peptone, 2 g yeast extract 35 g artificial sea salts and 0.5 g chloramphenicol; the pH was set to 8. 

The growth of the fungal cells in liquid cultures was traced by measuring optical density (OD) which determines the absorbance of a material and is a dimensionless quantity. The optical density was measured on the inoculation day (day zero) with the UV-1800 Shimadzu Spectrophotometer (λ = 660 nm, Kyoto, Japan) followed by regular checks of the OD to track the fungal growth stages.

To investigate the morphology of the fungal strains, the isolates were sampled during the exponential phase and fixed with formaldehyde (Sigma-Aldrich, 37%, St. Louis, MO, USA) to a final concentration of 2%. To dilute the fungal culture, 250 μL of the liquid culture were mixed with 5 mL MilliQ-water in an Eppendorf tube and subsequently filtered onto GTTP filters (0.22 µm, 25 mm diameter, Merck Millipore, Burlington, MA, USA). Afterwards, the sampling tube was flushed with 5 mL MilliQ-water and filtered again. The filter was dried and mounted on a microscopic slide. Two different methods were used for staining the fungal cells: Calcofluor-White and DAPI (4′,6-diamidino-2-phenylindole, a fluorescent stain that binds strongly to the adenine- thymine-rich regions in the DNA). To perform Calcofluor-White staining, a mixture of 25 μL of Calcofluor-White (a fluorescent dye that binds non-specifically to chitin and cellulose, obtained from Sigma-Aldrich in St. Louis, MO, USA) and 25 μL of 10% KOH solution (mixed with 10% glycerol) was prepared. This mixture was then added to the filter based on concentrations previously determined to yield the optimal signal intensity. To stain fungal cells with DAPI, 50 μL of DAPI-mix [5.5 parts of Citifluor (Electron Microscopy Sciences, Hatfield, PA, USA), one part of Vectashield (Vector Laboratories, Newark, CA, USA) and 0.5 parts of phosphate-buffered saline (PBS) with DAPI solution (final concentration 2 g/mL)] were used to stain the culture. The samples were analyzed with a Zeiss Axio Imager 2 microscope using UV-light below 400 nm (1000× magnification).

### 2.4. Physiological Tests of Fungal Growth

To investigate the fungal growth under various environmental conditions, liquid cultures of each species were prepared in biological triplicates per treatment (see below culture conditions). Samples were taken at regular intervals from the liquid cultures in order to measure the optical density (OD) as an indicator of the different stages of growth (Table 2). Sampling was discontinued once the cultures reached the stationary phase.

#### 2.4.1. Influence of Temperature

To study the effect of temperature on fungal growth, liquid media was prepared containing (g/L): 2 g glucose, 2 g peptone, 2 g yeast extract and 35 g artificial sea salts and 0.5 g chloramphenicol; the pH was set to 8. The liquid media was distributed in triplicate bottles per species. For inoculation of the liquid media, solid fungal biomass derived from the agar plates was mixed with MilliQ-water to a final OD of approximately one. Afterwards, 1 mL of this inoculum was added to 100 mL of liquid media. Finally, the bottles were incubated at 5 °C, 23 °C (room temperature) and at 35 °C on shaker incubators (Lab Companion, Shaking Incubator model ISS-7100R, 140 rpm, Billerica, MA, USA).

#### 2.4.2. Influence of Salinity

To study the effect of salinity on fungal growth, liquid media was prepared containing (g/L): 2 g glucose, 2 g peptone, 2 g yeast extract, 0.5 g chloramphenicol and either 0 g, 20 g, 35 g or 40 g of artificial sea salts. The liquid media was adjusted to a pH of 8. For each species, triplicate bottles were prepared and inoculated with a mix of MilliQ-water and fungal biomass as explained previously. Afterwards, the bottles were incubated at 23 °C (room temperature) on shaker incubators (Lab Companion, Shaking Incubator model ISS-7100R, 140 rpm, Billerica, MA, USA).

### 2.5. Metabolic Activity and Assimilation of Carbon Compounds by Fungal Cultures 

FF MicroPlate^TM^ (Biolog, Inc., Hayward, CA, USA) plates were used to investigate the species-specific carbon uptake of the fungal isolates by providing them with different carbon sources in a 96-well plate. These can give insight into the “Metabolic Fingerprint” of different fungal species. Carbon sources used in this study include a variety of compounds such as carbohydrates, amino acids, carboxylic acids, polymers, as well as others including alcohols, nucleotides, nucleosides, amines, and amides.

The culture inocula was prepared according to manufacturer guidelines and incubated on the 96 well plates at 23 °C in the dark. During this incubation period, the absorption was measured every day for four days at a wavelength of 490 nm and 750 nm with the Tecan M200 Infinite Pro (Männedorf, Swizerland). 

Increased mitochondrial respiration activity that results from oxidation of metabolizable carbon sources can be detected by the formation of a reddish-orange color at 490 nm. This is caused by the reduction in colorless tetrazolium chloride turning into red formazan [25]. The response in turbidity caused by the production of mycelia was measured at 750 nm. For our analysis, we used the values obtained on the fourth day of incubation, and as a result, these values are presented in the results.

### 2.6. Statistical Analysis

All statistical analyses were performed in R (version 3.6.1). The data was visualized with the package ggplot2 (version 3.3.5) [26]. In order to compare the utilization of different carbon compound groups by each species, a Kruskal–Wallis test was conducted. Subsequently, a pairwise comparison was carried out using the Wilcoxon test, and the resulting *p*-value was adjusted for multiple comparisons using the Bonferroni correction. The *p*-values were calculated with the package ggpubr (version 0.4.0). The statistical tests were performed with the package rstatix (version 0.7.0) [27]. Statistics in the text are depicted as mean ± standard deviation.

## 3. Results & Discussion

### 3.1. Isolated Fungal Species

The three different fungal isolates were identified as *Scheffersomyces spartinae, Sarocladium kiliense* and *Rhodotorula sphaerocarpa*, by the Westerdijk Fungal Biodiversity Institute. Additionally, we characterized all species physiologically by investigating their growth pattern under different salinities and temperatures as well as their preferred carbon substrate uptake.

**Figure 2:**
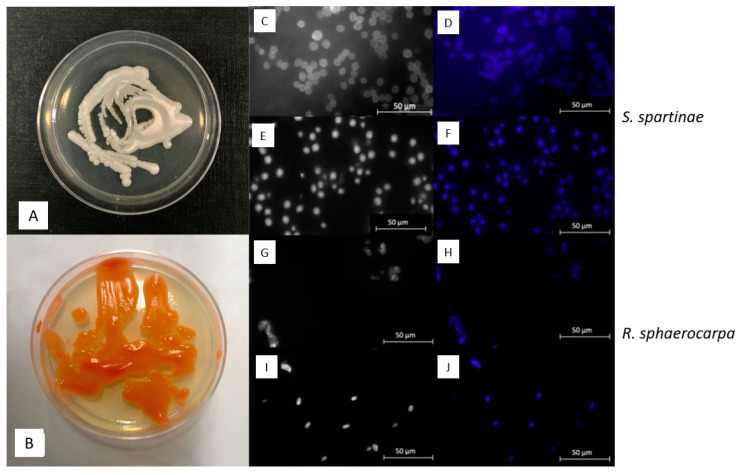
Phenotype of *Scheffersomyces spartinae* (**A**,**C**–**F**) and *Rhodotorula sphaerocarpa* (**B**,**G**–**J**). (**A**,**B**): Colonies after one week of growth; (**C**,**D**,**G**,**H**): Fungal cells stained with Calcofluor-White; (**E**,**F**,**I**,**J**): Fungal cells stained with DAPI-mix. Scale bar = 50 µm. Microscopic images were taken under UV-light below 400 nm.

The fungal ascomycetous yeast *Scheffersomyces spartinae* (Debaryomycetaceae, Saccharomycetales, Saccharomycetidae, Saccharomycetes, Saccharomycotina, Ascomycota) (Figure 2A,C–F) is exclusively known from aquatic environments. In previous studies, this species was isolated from habitats including brackish waters where it was found to be in association with marsh grass [28]. Additionally, it is recognized for its capacity to potentially produce coenzyme Q-9, which plays a role in the electron transport chain and is therefore of interest in the field of biomedicine [29]. Furthermore, *S. spartinae* has been shown to potentially act as bio-control agent [30,31].

**Figure 3:**
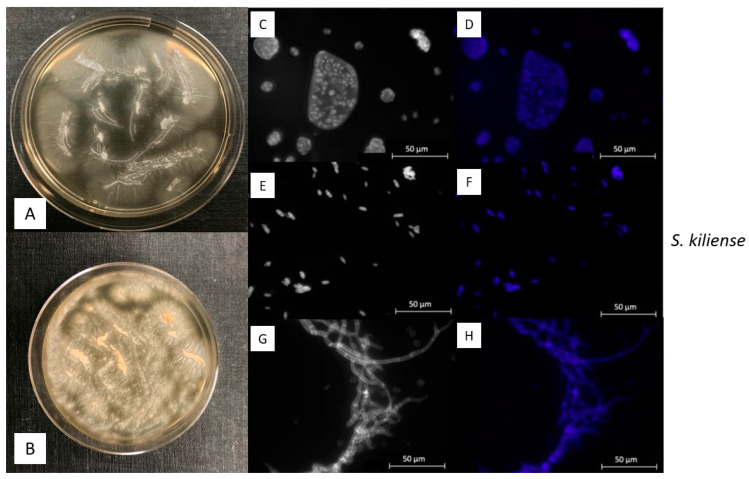
Phenotype of *Sarocladium kiliense*. (**A**): Colony after one week of growth; (**B**): Colony after four weeks of growth; (**C**,**D**): Fungal conidia stained with DAPI-mix; (**E**,**F**): Ellipsoidal Conidia stained with DAPI-Mix; (**G**,**H**): Fungal hyphae stained with Calcolfuor-White. Scale bar = 50 µm. Microscopic images were taken under UV-light below 400 nm.

*Sarocladium kiliense* (Figure 3) belongs to the division of Ascomycota (Hypocreales, Hypocreomycetidae, Sordariomycetes, Pezizomycotina) and grows as hyphae-morphotype. Although this taxon has a worldwide distribution, it is frequently found in soils and has been shown to be an opportunistic pathogen in humans [32]. It is described as being alkalotolerant, meaning that it can occur in both alkaline and acidic environments. However, it has been recently isolated from very alkaline environments with a pH of 10–12 [33]. Additionally, *S. kiliense* has been found in the deep sea at a depth of 5070 m [34] and has shown its potential in mercury bioremediation [35].

*Rhodotorula sphaerocarpa* (Sporidiobolaceae, Sporidiobolales, Incertae sedis, Microbotryomycetes, Pucciniomycotina, Basidiomycota) (Figure 2B,G–J) is an oleaginous (oil producing) yeast which occurs in typical pink-reddish colonies resulting from pigments that block certain wavelengths to prevent cell damage. The species has been isolated from diverse environments, including mangrove forests [36], hypersaline waters and salterns [37], and deep sea sediment [38].

### 3.2. Macroscopic and Microscopic Morphology of isolated Fungal Cultures

After one week of incubation at room temperature, the cells of *S. spartinae* showed thin branches, some with wobbly structures and a white coloration (Figure 2A,C–F). The coloration changed slightly after one month of incubation to a more yellowish tone. The shape of the cultures in general got flatter with edges being slightly more toothed. The cells of *S. spartinae* appeared to be mostly oval and ellipsoidal. They showed a rather uniform size between 1.5–2.2 × 4.1–5.3 µm (Figure 2C–F). The majority of cells were observed to occur individually, although some were found to be attached, indicating asexual reproduction through budding. In general, the morphological appearance of *Scheffersomyces spartinae* in the cultures closely resembled what had been reported in previous studies [28].

*S. kiliense* exhibited a relatively rapid growth and developed flat colonies being slightly raised in the center. After one week of incubation, the colony had a white to cream appearance (Figure 3A) which changed after around one month of incubation to a yellowish to light orange tone (Figure 3B). Apart from the color, this taxon showed a denser growth with furry structures. 

In general, the most common asexual reproductive spores of *S. kiliense* are called conidia and emerge from conidiophores or hyphae. In this study, we observed conidia accumulated around the tip of hyphae (Figure 3G,H). However, they also appeared dispersed (Figure 3E,F) and in slimy masses (Figure 3C,D). Single-celled conidia were shaped either oval or ellipsoidal with an average size between 3–5 × 2–3 μm (Figure 3E,F).

The colony of *R. sphaerocarpa* showed a bright color after about one week of growth with a coral to saffron orange coloration (Figure 2B). This appearance changed after approximately one month of incubation time at room temperature, after which the color changed to a lighter nuance of orange. In general, colonies of *R. sphaerocarpa* appeared shiny with a smooth structure. Like *S. spartinae*, the cells of *R. sphaerocarpa* occurred either individually or attached to each other, indicating active budding sites (Figure 2G–J).

In summary, the morphological characteristics of all species are similar to reports in previous studies [28,39] and confirm the species identification by parallel amplicon sequencing. 

### 3.3. Fungal Growth under Changing Environmental Conditions 

#### 3.3.1. Influence of Temperature on Fungal Growth

**Figure 4:**
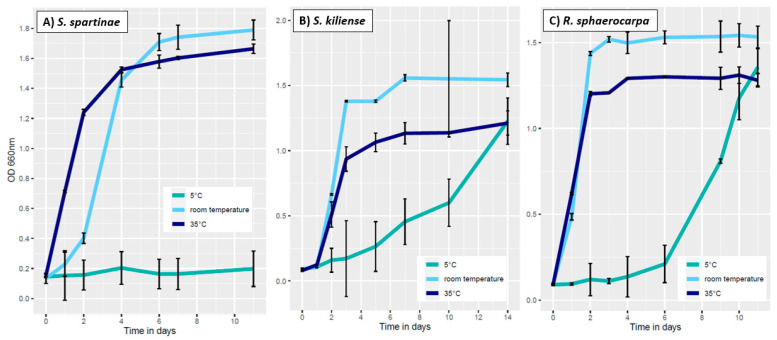
Growth curves at different temperatures (5 °C, room temperature 23 °C, 35 °C) for *Scheffersomyces spartinae* (**A**), *Sarocladium kiliense* (**B**) and *Rhodotorula sphaerocarpa* (**C**). Optical density (OD) refers to values at a wavelength of 660 nm. The average is shown, error bars indicate the standard deviation (SD).

The lowest temperature tested in this experiment was 5 °C in which the cells of *S. spartinae* never reached the exponential phase (Figure 4A). The OD values here ranged from 0.15 ± 0.01 to 0.19 ± 0.01. In contrast, *S. kiliense* entered the exponential phase on the seventh day of incubation (OD 0.45 ± 0.12), and the stationary phase on day 14 at 5 °C (Figure 4B). Similarly, the liquid culture of *R. sphaerocarpa* reached the exponential phase at day 9 (OD 0.81 ± 0.07), followed by the stationary phase on day 10 (OD 1.17 ± 0.05) (Figure 4C). Hence, although a general slower growth, both species show the ability to grow in colder waters. These findings are also in accordance with Nagahama [38] and Fan, et al. [34] who isolated *R. sphaerocarpa* and *S. kiliense*, respectively, from cold deep-sea environments. However, since the lowest growth yields of all species were obtained at 5 °C compared to the other temperatures, only a weak adaptation to cold waters can be assumed. Instead, the isolated strains seemed to be more adapted to temperatures close to their in situ conditions. 

At room temperature (23 °C), *S. spartinae* reached the exponential phase on the second day of incubation with an OD of 0.41 ± 0.08. On the fourth day, the fungal biomass increased to an OD of 1.46 ± 0.03, indicating the stationary phase (Figure 4A). Afterwards, the OD still increased until day 11 to a maximum OD of 1.79 ± 0.08. Additionally, *S. kiliense* reached the exponential phase at room temperature on the second day of incubation with an OD of 0.66 ± 0.01 (Figure 4B). On day three, the stationary phase was reached with an OD of 1.38 ± 0.01. *Rhodotorula sphaerocarpa* showed a similar growth at room temperature: the exponential phase was already reached at day 1 (OD 0.49 ± 0.01) (Figure 4C). On day 2, the value increased to an OD of 1.21 ± 0.01, revealing that the stationary phase was reached (Figure 4C) and indicating the capability of quick growth of *R. sphaerocarpa* at moderate temperatures. These results provide evidence that the most favorable growth conditions for all three fungal species were those at 23 °C due to their highest growth yields under these conditions.

The third temperature investigated in this experiment was 35 °C. *Scheffersomyces spartinae* already reached the exponential phase on day one with an OD of 0.71 ± 0.01 (Figure 4A). On the second day, the stationary phase was reached with an OD of 1.24 ± 0.16. *Sarocladium kiliense* reached the exponential phase at 35 °C on day two (OD 0.51 ± 0.09) (Figure 4B). The values increased until the stationary phase was reached on day 7 (OD 1.13 ± 0.08), indicating that this species can grow at warmer temperatures as well although with a lower total biomass at 35 °C in the stationary phase compared to *S. spartinae*. The fungal cells of *R. sphaerocarpa* reached the exponential phase on day one with an OD of 0.62 ± 0.01 (Figure 4C). On the second day, the fungal culture entered the stationary phase (OD 1.21 ± 0.09) with an intermediate maximum growth compared to the other two species (Figure 4); still indicative of good adaptations to warmer environmental conditions.

#### 3.3.2. Influence of Salinity on Fungal Growth

**Figure 5:**
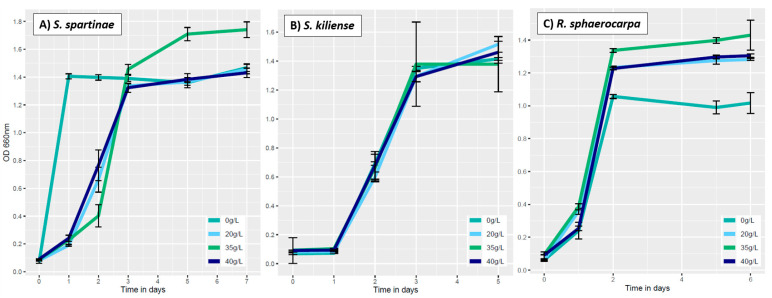
Growth curves at different salinities (0, 20, 35 and 40 g/L) for *Scheffersomyces spartinae* (**A**), *Sarocladium kiliense* (**B**) and *Rhodotorula sphaerocarpa* (**C**). Optical density (OD) refers to values at a wavelength of 660 nm. The average is shown, error bars indicate the standard deviation (SD).

The culture of *S. spartinae* incubated in the media that contained 0g/L of sea salts exhibited a steep increase in OD from 0.06 ± 0.01 to 1.41 ± 0.02 already after the first 24 h of incubation (Figure 5A). These values are quite remarkable since this species is not yet documented to occur in freshwater systems. However, our results indicate that it can also grow under non-saline conditions (Figure 5A). Yet, *S. spartinae* has been isolated from brackish waters, where the salinity is usually very low (0.1–1%) caused by the mixing of fresh- and seawater [28]. At 0 g/L sea salts, *S. kiliense* reached the exponential after one day of incubation (OD of 0.07 ± 0.00) and the stationary phase on the third day (OD of 1.35 ± 0.01) (Figure 5B). *Rhodotorula sphaerocarpa* started to grow exponentially during the first day of incubation and reached the stationary phase after two days with an OD of 1.06 ± 0.01 at a salinity of 0 g/L (Figure 5C). These results indicate the environmental flexibility of this species although it was previously mainly isolated from salty environments [36,37] but shows here that salts are not necessary for its successful growth. Still, compared to the other salinity treatments, *R. sphaerocarpa*’s total biomass was lowest at 0 g/L.

The second salinity concentration tested was 20 g/L. At this concentration, *S. spartinae* started to grow exponentially after one day (OD 0.19 ± 0.01) and reached the stationary phase after three days (OD 1.34 ± 0.03) (Figure 5A). Similarly, *S. kiliense* started to grow exponentially after one day (OD 0.08 ± 0.05) and reached the stationary phase on day three (OD of 1.29 ± 0.02) (Figure 5B). Additionally, for *R. sphaerocarpa*, the exponential growth started after one day of incubation (OD 0.36 ± 0.02) whereas the stationary phase was already reached on the second day (OD 1.23 ± 0.01), showing a quicker growth at 20 g/L salinity compared to the two other species (Figure 5C).

At a salinity concentration of 35 g/L, *S. spartinae* entered the exponential phase on day two with an OD of 0.40 ± 0.08 and the stationary phase on day five (OD 1.71 ± 0.05) (Figure 5A). *S. kiliense* reached the exponential after one day (OD of 0.1 ± 0.00) and the stationary phase on the third day (OD of 1.37 ± 0.29) (Figure 5B). *R. sphaerocarpa* started to grow exponentially after one day (OD 0.39 ± 0.02) and reached the stationary phase on the second day (OD 1.34 ± 0.01) (Figure 5C). Under these conditions, *S. spartinae* and *R. sphaerocarpa* exhibited the highest total biomass compared to the other salinity treatments. These results are consistent with the observation that the salinity concentration of 35 g/L reflects the normal range of ocean salinity where the fungi were originally isolated from, which lies between 34.9 g and 36 g per liter of seawater (Table 1).

In the media that contained 40 g/L of sea salts, the fungal cells of *S. spartinae* entered the exponential phase after one day of growth (OD 0.24 ± 0.02), and the stationary phase was reached after around three days (OD 1.32 ± 0.03) (Figure 5A). These final OD values of the stationary phase are comparable to that of the treatments of 0g/L and 20 g/L. This is not surprising, since a study conducted by Frigon and Liu [40] on wastewater treatment showed that *S. spartinae* could tolerate concentrations of salinities up to 120 g/L of NaCl. This emphasizes the potential wide tolerance towards different salinity conditions in this species. Under the same conditions, *S. kiliense* reached the exponential phase on day one with an OD value of 0.68 ± 0.01 and the stationary phase on the second day (OD of 1.29 ± 0.01) (Figure 5; B). In general, the culture of *S. kiliense* showed a very similar growth curve at all four salinity concentrations tested (Figure 5B). The fact that the fungus is not affected by changes in salinity aligns with its ubiquitous presence, as it has been found in marine environments [34], as well as in freshwater ecosystems such as highly alkaline lakes [33]. This suggests that the fungus has the ability to thrive in diverse aquatic environments despite changes in salt levels [33,34]. Additionally, at 40 g/L sea salts, *R. sphaerocarpa* exhibited a similar growth pattern compared to 20 g/L sea salts: i.e., after one day, the fungal culture started to grow exponentially (OD 0.25 ± 0.01) and reached the stationary phase on the second day (OD 1.23 ± 0.01) with final biomass concentrations after 6 days similar to 20 g/L sea salts (Figure 5C).

In conclusion, the change of salinity did not seem to have a strong effect on the growth of oceanic fungal cultures in general. Although some species exhibited a slightly steeper or less steep growth curve compared to others at a given salinity, our experiments proved the wide tolerance of the isolated fungal cultures to different environmental salinities. Similar results have been shown before [41] and again highlight the potential of pelagic fungi to adapt to future global warming of the oceans accompanied by changes in salinity.

### 3.4. Metabolic Activity (FF-Plates)

#### 3.4.1. Absorption at 750 nm (Fungal Growth)

*S. spartinae* utilized 89 of 95 provided carbon compounds whereas *S. kiliense* and *R. sphaerocarpa* utilized all 95 provided carbon compounds (Supplementary File S1). 

For *S. spartinae,* the three highest values were obtained for the utilization of carbon sources glycogen (1.81) (polymer), sedoheptulosan (1.74) (carbohydrate), and i-erythritol (1.56) (carbohydrate) (Supplementary File S1). The substrate types with the lowest fungal growth were D-trehalose (0.09) (carbohydrate), salicin (miscellaneous) (0.08) and palatinose (carbohydrate) (0.07). 

The fungus *S. kiliense* showed the highest OD values for the carbon sources glycerol (miscellaneous) (OD 2.11), N-acetyl-D-glucosamine (carbohydrate) (OD 2.07) and 𝛼-D-Glucose-1-Phosphate (carbohydrate) (OD 2.04) (Supplementary File S1). The substrate types showing the lowest fungal growth by this species were D-melezitose (carbohydrate) (0.24), turanose (carbohydrate) (0.22) and D-psicose (carbohydrate) (0.18). 

In comparison, the three substrates showing the highest growth of *R. sphaerocarpa* were L-lactic-acid (carboxylic acid) (OD 1.02) followed by D-galacturonic acid (carboxylic acid) (OD 0.9) and D-cellobiose (carbohydrate) (OD 0.84) (Supplementary File S1). The substrates with the lowest growth were amygdalin (miscellaneous) (OD 0.12) followed by lactulose (carbohydrate), D-fructose (carbohydrate), maltose (carbohydrate), D-arabitol and D-tagatose (carbohydrates), all with an OD of 0.14. The overall growth of *Rhodotorula sphaerocarpa* on the different substrates was lower than compared to the two other species; however, it could use all the provided carbon source for its growth. Furthermore, it also preferred more complex carboxylic acids for its growth compared to more simple carbohydrates which were favored by the other two species.

In this study we found a similar metabolic pattern between the three investigated species with highest fungal growth related mostly to carbohydrate-based substrates. Similar results were reported before [25], in which a saline-alkaline-tolerant fungal species was shown to metabolize more simple carbon sources. Although, at this point, research on the specific carbon substrate preferences of oceanic fungi is limited, these results give first implications about potential ecological niches in the cycling of certain carbon sources in planktonic food webs.

#### 3.4.2. Absorption at 490 nm (Fungal Respiration)

**Figure 6:**
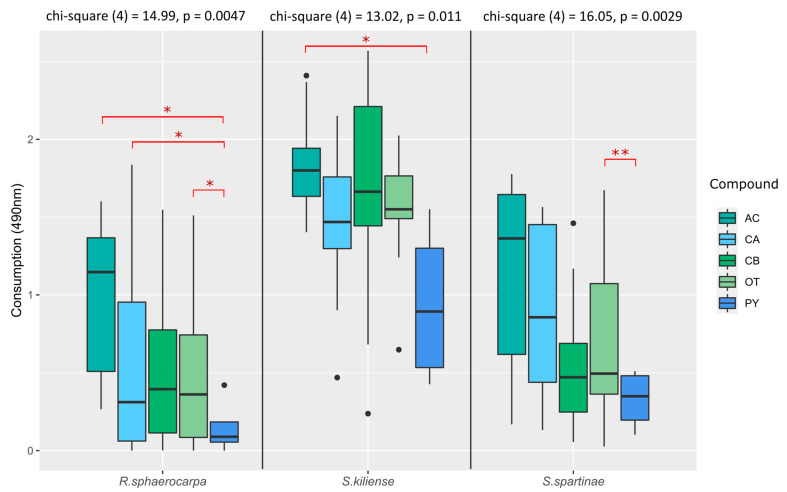
Utilization of different carbon compound groups by *Rhodotorula sphaerocarpa*, *Sarocladium kiliense* and *Scheffersomyces spartinae* cultures measured as absorption (OD) at a wavelength of 490 nm indicative of fungal respiration. The carbon compound groups are: AC- amino acids, CA- carboxylic acids, CB- carbohydrates, OT- others, PY- polymers. On top of each species-plot are the resulting statistics calculated with the Kruskal–Wallis test. The significance levels within each species-plot were calculated with pair-wise Wilcoxon test and the p-value adjusted with Bonferroni correction (* *p*-value < 0.05, ** *p*-value < 0.01, dots represent outliers).

In general, we found a tendency for the preferred utilization of amino acids (AC) followed by carboxylic acids (CA), carbohydrates (CB) and other carbon compounds (OT) whereas polymers (PY) were preferred the least (Figure 6). Significant differences between the preferred utilization of amino acids, carboxylic acids and “other” carbon compounds over polymers were found for *R. sphaerocarpa* (Figure 6). Additionally, significant differences between the utilization of amino acids and polymers were found in *S. kiliense* and between “other” carbon compounds and polymers in *S. spartinae* (Figure 6). This points out that more complex compounds might take more time to metabolize compared to simple carbon substrates which are being utilized faster within the first four days of incubation. Additionally, these results are similar to those reported by Nishida, et al. [42], who found that amino acids are a substantive nutritional source for fungi in general. Moreover, an experiment conducted by Shi, et al. [43] proved that the metabolic activity of *S. spartinae* can be enhanced by adding amino acids to the growth media.

A high variation in the consumption of carboxylic acid-compounds was found for *S. spartinae* (OD 0.93 ± 0.55) and *R. sphaerocarpa* (OD 0.66 ± 0.58) (Figure 6). However, compared to the two other species, *S. kiliense* showed on average the highest total mitochondrial activity for oxidizing carboxylic acids (OD 1.47 ± 0.46) (Figure 6). This is in line with a study by Fiebig [44], in which it was shown that aquatic fungi utilized carboxylic acids to a big extent from labile components of DOM (dissolved organic matter) and hence within the first days of incubation.

Furthermore, the results of the study indicated that *S. kiliense* had the highest metabolic activity for the utilization of carbohydrates (CB), with an average OD value of 1.75 ± 0.52. In comparison, *S. spartinae* had an average OD value of 0.51 ± 0.33 and *R. sphaerocarpa* had an average OD value of 0.5 ± 0.42 (Figure 6). Similar results were shown in a previous study where species of *Sarocladium* efficiently degraded carbohydrates such as polysaccharides, pectin, and precisely *S. kiliense* exhibited high degradation rates of starch [25].

The substrate category of “others” contained alcohols, nucleotides, nucleosides, amines and amides. Again, *S. kiliense* exhibited the highest metabolic activity of these substrates with an OD of 1.58 ± 0.30 (Figure 6). In contrast, *S. spartinae* had an OD of 0.36 ± 0.51 and *R. sphaerocarpa* of 0.50 ± 0.42. 

Lastly, in the group of polymers (PY), the metabolic activities of all species compared to the other compounds were lowest (*S. kiliense*: OD 0.94 ± 0.53; *S. spartinae*: OD 0.33 ± 0.2; *R. sphaerocarpa*: OD 0.2 ± 0.19), indicating that polymers are not oxidized to the same extent as the other substrates within the first 4 days of incubation. 

**Figure 7:**
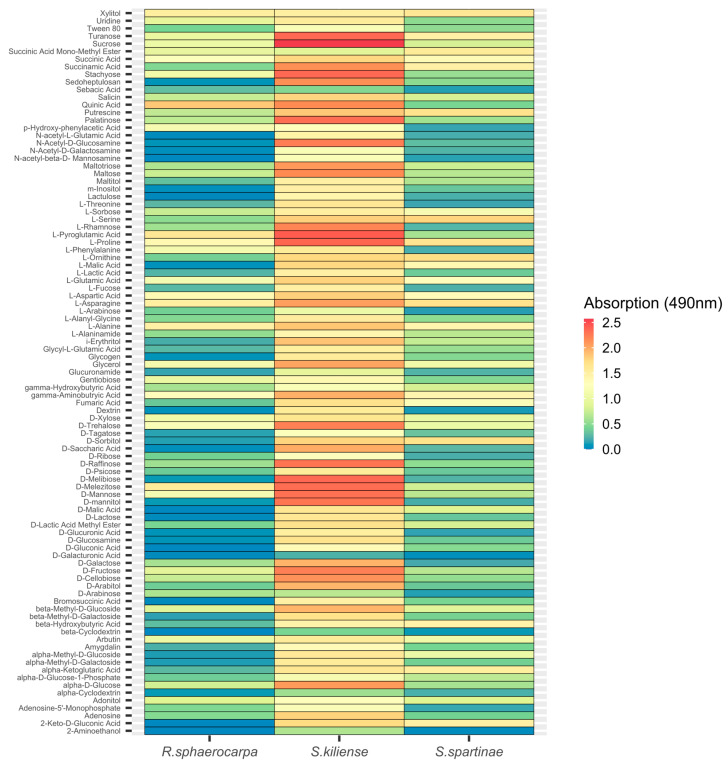
Utilization of 95 different carbon sources by *Rhodotorula sphaerocarpa*, *Sarocladium kiliense* and *Scheffersomyces spartinae* cultures measured as absorption (OD) at a wavelength of 490 nm indicative of fungal respiration.

Additionally, detailed analysis of the utilization of individual carbon substrates, indicated that although *S. kiliense* exhibited a higher respiratory activity compared to the two other species, there was no general pattern towards the preference of specific carbon compounds (Figure 7). Instead, each of the three fungal isolates exhibited species-specific utilization patterns (Figure 7), emphasizing the vast metabolic potential of oceanic fungi to degrade different carbon substrates.

## 4. Conclusions

We isolated and characterized three pelagic fungal strains from the open Atlantic Ocean belonging to three different species. Despite being phylogenetically diverse, we observed shared physiological characteristics among all of the species. Specifically, we found that all of the isolated cultures displayed their highest total biomass values during the stationary phase at 23 °C, which is the temperature closest to the in situ conditions, when compared to other temperatures that were investigated. Yet, all species were able to grow successfully at elevated temperature (35 °C), which is especially relevant in future ocean warming scenarios. In spite of contrasting conditions at reduced temperature, two of the three species still achieved similar final biomass concentrations as in the warmest conditions tested. This suggests that these species have the potential to adapt to temperature changes in a range of 30 °C. Clearly, these results are species specific but suggest that changes in temperature through climate change could potentially have unpredictable impacts on marine fungal communities. 

Furthermore, the studied species seem to be very tolerant regarding the salinity concentration of their growth medium (much wider than the salinity range generally observed in the open ocean waters), indicating a remarkable capability for pelagic oceanic fungi to thrive in a vast range of salinities. Nevertheless, *S. spartinae* and *R. sphaerocarpa* had the highest growth yields in the medium with a concentration of 35 g/L sea salts, which is closest to their in situ oceanic conditions. 

The investigations at 750 nm of the FF-plates (indicative of fungal growth) showed that all fungal species showed highest growth on specific carbohydrates or carboxylic acids, which could be expected since simple and labile compounds are usually promptly degraded more easily. Similar results were found at 490 nm (indicative of fungal respiration) but in general, depending on species, substrate groups containing amino acids and carbohydrates displayed the highest metabolic activity across all the species. However, our results indicate that *S. kiliense* showed higher oxidation rates of all tested compound groups, which suggests potential differences in nutrient cycling among pelagic species. 

As mentioned above, marine fungi are active players in the oceanic elemental cycles. However, our knowledge on the specific roles played by individual fungal species in the ocean is very limited. Hence, it becomes a relevant task to investigate species-specific physiological characteristics and capabilities of marine pelagic fungi to better understand the role of the individual fungal members, populations, and communities in the marine ecosystem. Research such as this is valuable for gaining insight into the physiological characteristics of marine fungi and can help enhance our understanding of their ecology and distribution in the oceanic water column. 

## Figures and Tables

**Table 1:** Fungal species isolated during the ANTOM-1 and Poseidon Cruise.

ID	Species	Cruise	Depth	Temperature	Salinity	Location
ANTOM-1 Isolate 13	*Scheffersomyces spartinae*	ANTOM-1	5 m	27 °C	34.9	Latitude: 7.343 Longitude: ™29.581
ANTOM-1 Isolate 15	*Sarocladium kiliense*	ANTOM-1	45 m	26 °C	36	Latitude: 0.137 Longitude: ™30.501
Poseidon isolate	*Rhodotorula sphaerocarpa*	Poseidon	93 m	20 °C	35	Latitude: 22.220 Longitude: ™26.633

**Table 2:** Estimation of the fungal growth phase according to the optical density (OD).

Growth Phase	Optical Density (660 nm)
Adaptation	0.070 to 0.399
Exponential	0.400 to 1.199
Stationary	>1.200

## Data Availability

The raw data supporting the conclusions of this article will be made available by the authors, without undue reservation to any qualified researcher.

## Supplementary Materials

The following are available online at https://www.mdpi.com/article/10.3390/jof9040439/s1, Supplementary File S1: Utilization of carbon substrates by *Rhodotorula sphaerocarpa*, *Sarocladium kiliense* and *Scheffersomyces spartinae* cultures measured as absorption (OD) at a wavelength of 750 nm indicative of fungal growth.
